# In vitro and computational investigation of antioxidant and anticancer properties of *Streptomyces coeruleofuscus* SCJ extract on MDA-MB-468 triple-negative breast cancer cells

**DOI:** 10.1038/s41598-024-76200-8

**Published:** 2024-10-24

**Authors:** Said Rammali, Abderrazak Idir, Marouane Aherkou, Alin Ciobică, Fatima Zahra Kamal, Mohamed El Aalaoui, Abdellatif Rahim, Abdelkrim khattabi, Zyad Abdelmajid, Abderrahim Aasfar, Vasile Burlui, Gabriela Calin, Ioannis Mavroudis, Bouchaib Bencharki

**Affiliations:** 1 Laboratory of Agro-Alimentary and Health, Faculty of Sciences and Techniques, Hassan First University of Settat, B.P. 539, Settat, 26000 Morocco; 2 Agro-Industrial and Medical Biotechnology Laboratory, Team of Experimental Oncology and Natural Substances, Faculty of Sciences and Technology, Sultan Moulay Slimane University, Beni-Mellal, Morocco; 3 Science and Technology Team, Higher School of Education and Training, Chouaîb Doukkali University, El Jadida, Morocco; 4 Mohammed VI University of Sciences and Health, Casablanca, Morocco; 5 Mohammed VI Centre for Research and Innovation (CM6RI), Casablanca, Morocco; 6 Biotechnology Laboratory (MedBiotech), Bioinova Research Center, Faculty of Medicine and Pharmacy, Mohammed V University, Rabat, Morocco; 7 Department of Biology, Faculty of Biology, Alexandru Ioan Cuza University of Iasi, 20th Carol I Avenue, Iasi, 700506 Romania; 8 Center of Biomedical Research, Iasi Branch, Romanian Academy, Teodor Codrescu 2, Iasi, 700481 Romania; 9 Academy of Romanian Scientists, 3 Ilfov, Bucharest, 050044 Romania; 10 Higher Institute of Nursing Professions and Health Technical (ISPITS), Marrakech, 40000 Morocco; 11 Laboratory of Physical Chemistry of Processes and Materials, Faculty of Sciences and Techniques, Hassan First University, Settat, 26000 Morocco; 12 Regional Center of Agronomic Research of Settat, Tertiary Road 1406, At 5 Km from Settat, Settat, 26400 Morocco; 13 Laboratory of Biochemistry, Neurosciences, Natural Ressources and Environment, Faculty of Sciences and Techniques, Hassan First University of Settat, B.P. 539, Settat, 26000 Morocco; 14 Plant and Microbial Biotechnology center, Moroccan Foundation for Advanced Science, Innovation and Research (MAScIR), Mohammed VI Polytechnic University, Ben Guerir, Morocco; 15 “Ioan Haulica Institute”, Apollonia University, Păcurari Street 11, Iasi, 700511 Romania; 16 Leeds Teaching Hospitals, NHS Trust, Leeds, LS97TF UK

**Keywords:** *Streptomyces coeruleofuscus*, Antioxidant activity, Anticancer activity, Triple-negative breast cancer, Pharmacokinetic properties, HPLC-UV/vis, Biochemistry, Biological techniques, Microbiology

## Abstract

This study aimed to explore the antioxidant potential of the ethyl acetate extract of *Streptomyces coeruleofuscus* SCJ strain, along with its inhibitory effects on the triple-negative human breast carcinoma cell line (MDA-MB-468). The ethyl acetate extract’s total phenolic and flavonoid contents were quantified, and its antioxidant activity was investigated using DPPH (1,1-Diphenyl-2-picrylhydrazyl), ABTS (2,2’-azino-bis (3-ethylbenzothiazoline-6-sulphonic acid), and FRAP (Ferric Reducing Antioxidant Power) assays. Furthermore, the cytotoxic effect of the organic extract from *Streptomyces coeruleofuscus* SCJ on MDA-MB-468 cancer cells was assessed via the crystal violet assay. In tandem, a thorough computational investigation was conducted to explore the pharmacokinetic properties of the identified components of the extract, utilizing the SwissADME and pKCSM web servers. Additionally, the molecular interactions between these components and Estrogen Receptor Beta, identified as a potential target, were probed through molecular docking studies. The results revealed that ethyl acetate extract of SCJ strain exhibited remarkable antioxidant activity, with 39.899 ± 1.56% and 35.798 ± 0.082% scavenging activities against DPPH and ABTS, respectively, at 1 mg/mL. The extract also displayed significant ferric reducing power, with a concentration of 1.087 ± 0.026 mg ascorbic acid equivalents per mg of dry extract. Furthermore, a strong positive correlation (*p* < 0.0001) between the antioxidant activity, the polyphenol and the flavonoid contents. Regarding anticancer activity, the SCJ strain extract demonstrated significant anticancer activity against TNBC MDA-MB-468 cancer cells, with an inhibition percentage of 62.76 ± 0.62%, 62.67 ± 0.93%, and 58.07 ± 4.82% at 25, 50, and 100 µg/mL of the extract, respectively. The HPLC-UV/vis analysis revealed nine phenolic compounds: gallic acid, sinapic acid, *p-*coumaric acid, cinnamic acid, trans-fereulic acid, syringic acid, chloroqenic acid, ellagic acid, epicatechin. *Streptomyces coeruleofuscus* SCJ showed promise for drug discovery, exhibiting antioxidant and anticancer effects.

## Introduction

Oxidative stress is a fundamental biological phenomenon resulting from an imbalance between antioxidants and the production of ROS^1^. These ROS are natural byproducts of routine cellular processes, primarily originating from activities in the mitochondria and enzymatic reactions^2^. It was reported that an overabundance of ROS or a decrease in antioxidant capacity can lead to oxidative stress. In certain cases, ROS can harm vital biomolecules like lipids, proteins, nucleic acids, and carbohydrates, potentially disrupting cellular functions and contributing to the development of various diseases^3^.

Breast cancer is the most common cancer among women worldwide^4^. When detected in its early stages without spreading to other parts of the body, it can be curable in approximately 70–80% of cases. However, for advanced breast cancer with distant organ metastases, current treatments are typically unable to offer a remedy^4,5^. Several factors, mainly environmental, hormonal, and infectious, are especially relevant in the progression of this tumor^6^. These factors are strongly related to the generation of ROS, which are involved in initiating DNA damage^7^, promoting cancer-related processes, and enhancing cell proliferation and survival^8^. Different molecular subtypes of breast cancer may respond uniquely to oxidative stress. For instance, oxidative stress can induce mutations or modifications in estrogen receptor (ER)-positive breast cancer^9^. Hence, it becomes crucial to introduce and execute innovative approaches for anticancer, and antioxidant interventions, in order to effectively address the aforementioned challenges.

TNBC is a type of breast cancer characterized by the absence of hormone receptors (estrogen (ER) and progesterone (PR)) and the lack of overexpression of human epidermal growth factor 2 (HER2)^10^. Due to the absence of these hormone receptors and the HER2 protein, breast cancers tend to be more aggressive than other types of breast cancer^11^. They are characterized by low differentiation, high invasiveness, propensity for local and distant metastases, poor prognosis due to their aggressiveness, and high regeneration rate^12,13^. The TNBC presents a greater presence of infiltrating lymphocytes, creating an immunological milieu conducive to the administration of immune checkpoint inhibitors (ICIs)^12^. TNBC also has a high mutational load, providing an antigenic basis for immune cell recognition^12^.

Recent studies have increasingly focused on exploring microorganisms from diverse habitats to discover novel compounds with a wide range of biological activities^14,15^. Among these, *Actinobacteria*, particularly the *Streptomyces* genus, have been found to possess significant medicinal properties. Beyond their well-known role in antibiotic production, *Streptomyces* and other *Actinobacteria* generate secondary metabolites that serve as potent anticancer, antioxidant, antifungal, and antiparasitic agents, demonstrating effectiveness against numerous pathogens and finding extensive use in clinical applications^15–17^. Remarkably, *Streptomyces*-derived antibiotics account for nearly 50% of all therapeutically valuable antibiotics, and each strain has the capacity to produce over 30 secondary metabolites on average^18,19^. In this study, the main objective was to evaluate the antioxidant activity of ethyl acetate extract derived from the *Streptomyces coeruleofuscus* SCJ strain, and to study the inhibitory effect of this extract on the triple-negative human breast carcinoma cell line (MDA-MB-468), as well as to predict the pharmacokinetic properties, toxicity and molecular interactions of volatile compounds extracted from *Streptomyces coeruleofuscus* SCJ, using in silico approaches, in order to provide valuable information for potential drug development, with a focus on the treatment of triple-negative breast cancer (TNBC).

## Materials and methods

### Actinobacteria used

The *Streptomyces coeruleofuscus* SCJ (NCBI GenBank Acc. No: OP101646) was isolated from a soil sample collected from the Northwestern Moroccan site located in Titt Mellil garden belonging to the Casablanca-Settat region of Morocco (33°33’14.1"N, 7°29’05.4"W), during February and early March 2019. In a recent study conducted in our Laboratory, it was demonstrated that the ethyl acetate extract of this strain exhibits highly significant antimicrobial activity against multi-resistant bacteria and phytopathogenic fungi. Additionally, GC-MS analysis of this extract revealed the presence of 9 volatile compounds, primarily 3,5-dimethylpyrazole and pyrrolizidine derivatives (such as Pyrrolo[1,2-a]pyrazine 1,4-dione, hexahydro-3-(2-methylpropyl))^18^, as documented in the literature for their potent anticancer and antioxidant activities^20–23^.

### Production and extraction of bioactive molecules

Bioactive molecule production and extraction followed a method adapted from Aouiche et al. (2014)^24^. Briefly, the *Streptomyces coeruleofuscus* SCJ underwent fermentation and subsequent extraction of secondary metabolites using ethyl acetate. Erlenmeyer flasks (500 mL) with 100 ml of ISP2 culture medium were inoculated with the strain and incubated at 28 °C with constant agitation (150 rpm). After centrifugation at 10,000 g for 20 min to remove mycelia, the supernatants were mixed with an equal volume of ethyl acetate in a separating funnel. The resulting organic extracts were evaporated at 45 °C to remove ethyl acetate. Dry organic extracts and residual aqueous phases were stored at 4 °C for future use.

### Determination of total phenolic contents

The quantification of total phenolic of the organic extract of *Streptomyces coeruleofuscus* SCJ was conducted using the Folin-Ciocalteu method^25^. Absorbance was measured at 760 nm. The total phenolic contents were expressed as mg gallic acid equivalents (GAEs) per mg of dry extract.

### Determination of flavonoid contents

The quantification of flavonoid in *Streptomyces coeruleofuscus* SCJ organic extract was performed by the aluminum trichloride method^26^. The absorbance was read at 415 nm. The total flavonoid compounds were expressed as mg quercetin (QEs) equivalents (GAEs) per mg of dry extract.

### In vitro antioxidant activity

#### DPPH free radical scavenging activity

The DPPH free radical scavenging activity of organic extract from *Streptomyces coeruleofuscus* SCJ was conducted following the method reported by Blois (1958)^28^. The crude extract of *Streptomyces coeruleofuscus* strain SCJ was diluted in absolute ethanol to make concentrations ranging from 0.1 to 1 mg/ml. In a 96-well microplate, 40 µL of the crude extract solutions at different concentrations were combined with 160 µL of DPPH (0.1 mM in methanol). The reaction mixture was agitated for 10 s and then incubated in darkness for 30 min at room temperature. Following incubation, absorbance was measured at 517 nm using an Elisa microplate reader (2100-C, Optic Ivymen Systems, Spain). Ascorbic acid was employed as a positive antioxidant control. The percentage of DPPH free radical scavenging by the extracts was determined using the following equation:

$$\:DPPH\:free\:radical\:scavenging\:activity\:\left(\%\right)=\:\left[\frac{\left(A0-A1\right)}{\left(A0\right)}\right]\times\:\:100$$

A_0_: Absorbance of blank.

A_1_: Absorbance of sample.

#### ABTS free radical scavenging activity

The ABTS free radical scavenging activity of organic extract from *Streptomyces coeruleofuscus* SCJ was assessed following the method reported by Re et al.^29^. Briefly, ABTS+-cationic radicals were generated by mixing 7 mM ABTS (dissolved in distilled water) with 2.45 mM potassium persulfate (dissolved in distilled water) and allowing the mixture to stand overnight in darkness. The concentrated ABTS+- solution was diluted with ethanol to achieve an absorbance of 0.7 at 734 nm using a spectrophotometer (Selectra VR2000, Barcelona, Spain). To 100 µL of each sample concentration (0.1 to 1 mg/mL), 2 mL of the diluted ABTS+- solution was added. Triplicate measurements were performed for each sample. Trolox was employed as a positive antioxidant control. The ABTS^+−^ free radical scavenging activity of the extracts was determined using the DPPH assay formula.

#### Ferric reducing antioxidant power (FRAP) test

The iron-reducing activity was assessed following the method reported by Oyaizu (1986)^30^. briefly, 500 µL of each sample concentration of extract (0.1 to 1 mg/mL) was mixed with 1.25 mL of phosphate buffer (0.2 M, pH 6.6) and 1.25 mL of a 1% aqueous solution of potassium hexacyanoferrate K3 [Fe (CN)_6_] within a test tube (15 mm × 125 mm). The resulting solution was incubated in a water bath at 50 °C for 20 min. Subsequently, 1.25 mL of trichloroacetic acid (10%) was added to stop the reaction, followed by centrifugation of the tubes at 3000 rpm for 10 min. A 1.25 mL portion of the supernatant was mixed with 1.25 mL of distilled water and 0.5 mL of a 0.1% FeCl_3_ aqueous solution. Absorbance was measured at 700 nm against a blank prepared in the same manner, replacing the extract with distilled water. As a positive standard, ascorbic acid was employed.

### Evaluation of anti-cancer activity

#### Cell culture and cytotoxicity

The MDA-MB-468 triple-negative human breast cancer cell line, “provided by the Experimental Oncology and Natural Substances Team at the Faculty of Sciences and Technology, Sultan Moulay Slimane University in Beni-Mellal, Morocco”, was cultivated in RPMI 1640 medium. The medium was enriched with 5% heat-inactivated fetal calf serum, 1% Penicillin-Streptomycin, and 0.2% L-glutamine for optimal growth conditions. Cell incubation took place in a humidified incubator at 37 °C and 5% CO_2_. The cytotoxic effect of the organic extract from *Streptomyces coeruleofuscus* SCJ on cancer cells was assessed using the crystal violet assay^31^. Approximately 10^4^ cells per well were seeded in a 96-well microplate containing 100 µL of complete culture medium. The microplate was then placed in an incubator (37 °C, 5% CO_2_) for at least 6 h. Following this, the cells were exposed to escalating concentrations of the extract (ranging from 0.78 to 100 µg/mL) and were maintained under identical conditions for 24 h. Post-incubation, the culture medium in each well was replaced with 70 µL of 5% crystal violet solution. After 30 min, excess crystal violet was removed, and a low-flow tap wash was performed. Subsequently, 100 µL of 1% SDS was added to each well, and the optical density was measured at 540 nm using a plate reader.

For assessment, three experimental repetitions were carried out, using DMSO as the negative control and paclitaxel as the positive control. The percentage of cell lysis was calculated based on the provided formula.

$\:Cell\:viability\:\left(\%\right)\:=100\:\times\:\left(\frac{A}{{A}_{0}}\right)$ A: Absorbance of the samples (cells treated by the extract).

A_0_: Absorbance of the negative control.

#### HPLC-UV/visible analysis

The HPLC-UV/visible analysis was conducted using a Shimadzu HPLC system with an SPD-20 A UV/absorbance detector. Separation occurred on a Waters reverse-phase Symmetry C-18 column (150 × 3.9 mm, 5 μm) at room temperature. The mobile phase consisted of deionized water with trifluoroacetic acid (TFA) (pH 2.5) as solvent A, and 99.99% methanol as solvent B. A gradient elution was employed: 100–50% solvent A from 0 to 20 min, 50–40% solvent A from 20 to 30 min, and 40–100% solvent A from 30 to 40 min. The flow rate was set to 1 mL/min, and the detector was adjusted to 280 nm^32^. Phenolic compounds were identified by comparing their retention times and UV-Vis spectra to those of reference standards, including gallic acid, vanillin, fereulic acid, vanilic acid, sinapic acid, *p*-coumaric acid, caffeic acid, cinammic acid, quercitin, trans-fereulic acid, syringic acid, chloroqenic acid, ellagic acid, epicatechin, and resornisol^32^.

### ADMET analysis

The nineteen compounds identified in the ethyl acetate crude extract of *Streptomyces coeruleofuscus* SCJ^33^, were used to conduct in silico analysis.

The canonical SMILES of these compounds were obtained from the PubChem database (https://www.pubchem.ncbi.nlm.nih.gov/) and employed in subsequent computational analysis.

The pharmacokinetic properties (Absorption, Distribution, Metabolism, and Excretion) and toxicity (ADMET) of these compounds were assessed using the pkCSM (https://biosig.lab.uq.edu.au/pkcsm/) and SwissADME (http://www.swissadme.ch//) web servers. Additionally, the potential cellular targets of these compounds were predicted using the Swiss Target Prediction server (https://www.swisstargetprediction.ch).

### Molecular docking analysis

The 3D structures of the specific compounds present in the ethyl acetate crude extract of *Streptomyces coeruleofuscus* SCJ were obtained from PubChem (source: https://pubchem.ncbi.nlm.nih.gov/). The SDF format of the ligands was imported into Discovery Studio version 4.5 to generate a library of ligands in PDB format.

The three-dimensional crystal structure of the selected estrogen receptor Beta (PDB ID: 7XWQ) was retrieved in PDB format from the RCSB protein database (source: https://www.rcsb.org).

PyMoL 2.5.3 was used to eliminate water molecules and solvents, as part of the preparation of proteins for molecular docking. Non-polar hydrogen atoms and Kollmann charges were then added by AutodockTools^34^.

To estimate binding strength, the Assisted Molecular Docking (AMDock) graphical interface was used to perform molecular docking using AutoDock Vina. a grid box was established, based on the binding site of the co-crystallized ligand. All other docking parameters were kept at their default values. The results of the docking analysis were examined using PyMol and 2D interaction visualizations were generated using the academic version of Maestro.

### Statistical analysis

Data from all experiments were repeated at least twice and results were expressed as Mean ± standard deviation. Ordinary one-way ANOVA followed by Tukey’s multiple comparisons test was performed using GraphPad Prism 8.4.3 software (GraphPad software Inc., San Diego, CA, USA) to test for significant differences between groups in phenolic and flavonoid contents as well as antioxidant and anticancer activities. The results were considered statistically significant when *p* < 0.05.

## Results and discussion

### Isolation and identification of *Streptomyces coeruleofuscus* SCJ

**Fig. 1:**
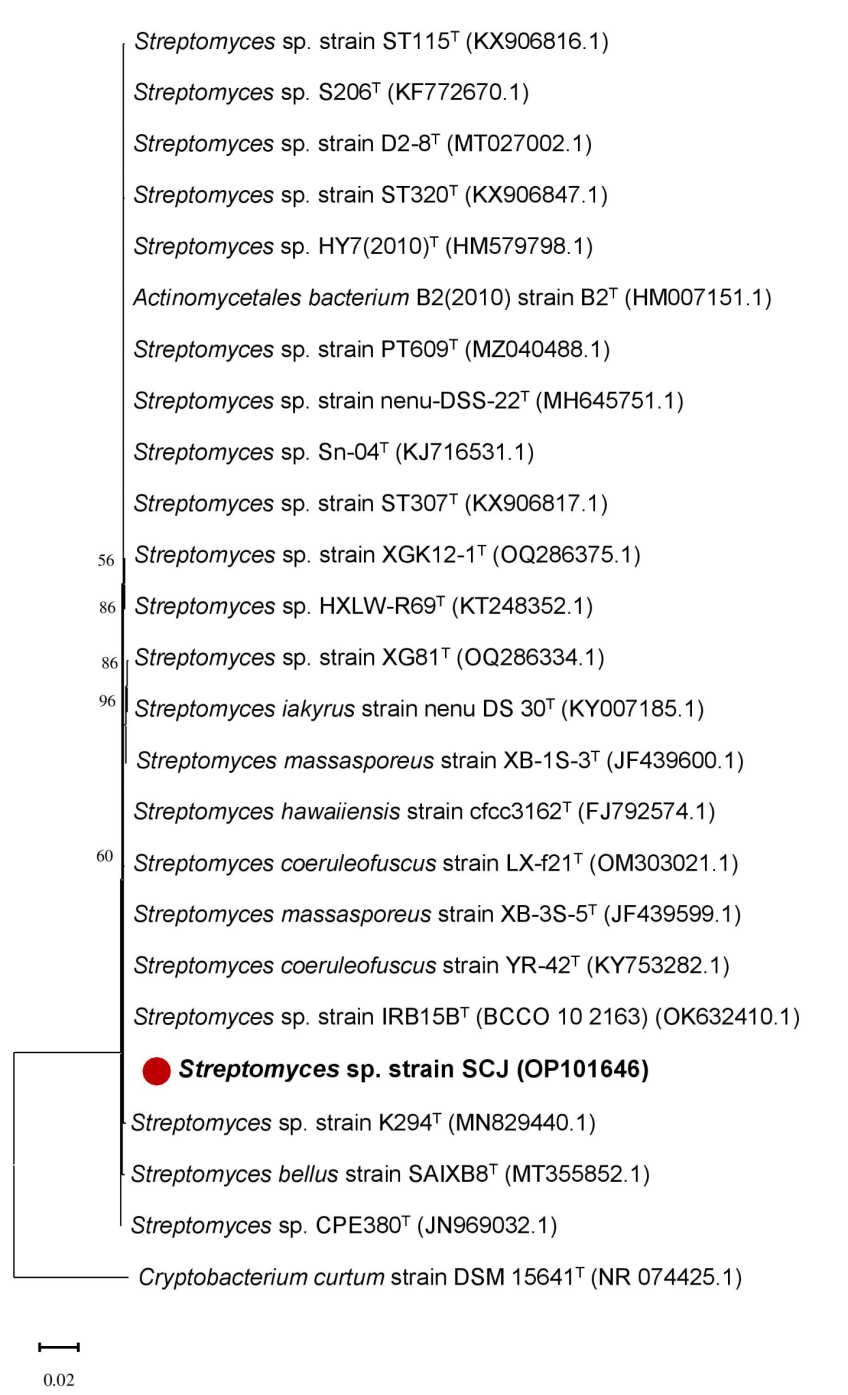
Phylogenetic tree (16 S rRNA gene) showing the evolutionary relationship between *Streptomyces* sp. SCJ and its closest known taxa using MEGA X. The bar (0.02) represents the number of substitutions per nucleotide position (1% divergence between sequences). The tree was generated using 1000 bootstraps and GenBank accession numbers are enclosed in parentheses. *Cryptobacterium curtum* was the outgroup in the analysis. T = Type strain.

The identification of new isolates with significant biological activities holds paramount importance. Consequently, the isolation of *Actinomycetes*, particularly from unexplored sites, is receiving worldwide attention. The Garden of Titt Mellil, located in the Casablanca-Settat region of Morocco, was chosen as a source for *Actinomycetes* isolation^18^. Among the isolates obtained, one stood out due to its ability to produce a violet-brown pigment that diffuses. This isolate was identified through 16 S rRNA sequencing as *Streptomyces coeruleofuscus* SCJ, and its sequence has been deposited in the GenBank of the National Center for Biotechnology Information (NCBI) under accession number OP10164618^33^(Fig. 1)^33^.

### Correlation between total phenolic and flavonoid contents and in vitro antioxidant activity of ethyl acetate extract of strain SCJ

Recent studies confirm that free radicals are pivotal in the development of diseases like cancer, autoimmune disorders, cardiovascular issues, and neurodegenerative disorders^35^. In addition, Yau & Benz^36^ has demonstrated that oxidative stress could contribute to the progression of breast cancers characterized by estrogen receptor positivity (ER-positive), including those with an ER-positive/PR-negative clinical phenotype (progesterone receptor negative)^36^. On the other hand, antioxidants are compounds that help protect the human body against oxidative stress by neutralizing the presence of free radicals that can damage cells, proteins and DNA^37^. These antioxidants therefore promote human health by preventing or mitigating the risk of diseases like cancer^37,38^.

The results of the total phenolic and flavonoid contents of the ethyl acetate extract of *Streptomyces coeruleofuscus* SCJ are summarized in Table 1. Pearson’s statistical analysis uncovered a positive correlation between varying concentrations of ethyl acetate extract and total phenolic contents (*r* = 0.9979; *p* < 0.001), along with a positive correlation with flavonoid contents (*r* = 0.9945; *p* < 0.0001). *Streptomyces* bacteria are known for producing natural compounds like phenolic and flavonoid compounds with demonstrated antioxidant properties and free radical scavenging abilities^39–41^. Notably, phenolic compounds such as phenol, 2,4-bis(1,1-dimethylethyl)^40^, phenol, 3,5-dimethoxy^40^, phenol^15^, and maltol^15^ have been identified in various *Streptomyces* strains, including *Streptomyces cavouresis* KUV39^42^, *Streptomyces* sp. MUM256^43^, *Streptomyces colonosanans*^44^, and *Streptomyces* sp. E23-4^15^. These compounds have shown significant antioxidant activity, which may contribute to the bioactivity of *Streptomyces* extracts. Although these specific compounds were not identified in our study, their presence in other similar strains suggests that analogous molecules could be responsible for the antioxidant effect observed in the *Streptomyces coeruleofuscus* extract. Similarly, the ethyl acetate extract from *Streptomyces coeruleofuscus* SCJ in this study displayed elevated levels of total phenolic and flavonoid compounds, as well as high in vitro antioxidant activity indicating its potential as a source of potent antioxidants to combat oxidative stress^45,46^.

**Table 1:** Total phenolic and flavonoid contents of ethyl acetate extract of *Streptomyces coeruleofuscus* SCJ. (ND): not determined, (GAE): gallic acid equivalent (QE): quercetin equivalent.

Concentration of ethyl acetate extract (mg/mL)	Total phenolic contents (mg GAE/mg extract	Total flavonoid contents (mg QE/mg extract)
0.1	0.308 ± 0.002	ND
0.2	0.338 ± 0.005	ND
0.3	0.351 ± 0.008	0.020 ± 0.005
0.4	0.395 ± 0.004	0.059 ± 0.011
0.5	0.429 ± 0.003	0.075 ± 0.010
0.6	0.453 ± 0.006	0.107 ± 0.004
0.7	0.494 ± 0.006	0.127 ± 0.006
0.8	0.519 ± 0.004	0.153 ± 0.004
0.9	0.551 ± 0.007	0.180 ± 0.005
1	0.591 ± 0.009	0.204 ± 0.006

**Fig. 2:**
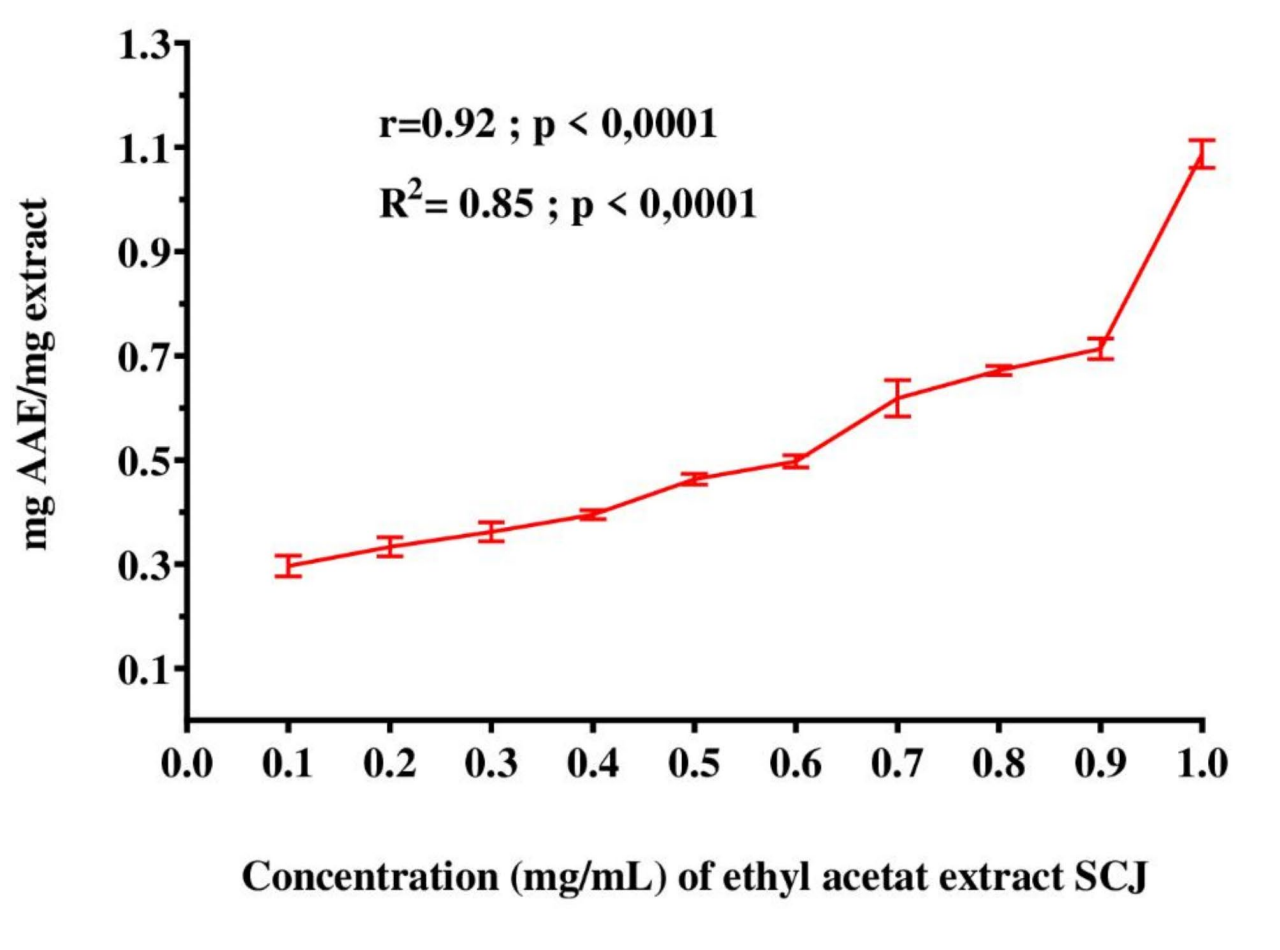
Ferric reducing antioxidant power (FRAP) assay of ethyl acetate extract of *Streptomyces coeruleofuscus* SCJ. Values expressed are means ± SD (*n* = 3). AAE: ascorbic acid equivalent.

According to Table 2, our findings revealed that at a concentration of 1 mg/mL, the ethyl acetate extract of strain SCJ exhibited a DPPH free radical scavenging activity of 39.899 ± 1.560%. Additionally, the extract displayed significant antioxidant activity in the ABTS assay, with a free radical scavenging activity of 35.798 ± 0.082% at the same concentration **(**Table 2**)**. Furthermore, the ethyl acetate extract of *Streptomyces coeruleofuscus* SCJ displayed significant FRAP activity, ranging from 0.296 ± 0.019 to 1.087 ± 0.026 mg ascorbic acid equivalents (EAA) per mg of extract dry weight, indicating strong antioxidant potential **(**Fig. 2**)**.

**Table 2:** Antioxidant activities of ethyl acetate extract of *Streptomyces coeruleofuscus* SCJ assessed using different antioxidant assays (ABTS and DPPH).

Concentrations (mg/mL)	Antioxidant activities			
	SCJ ethyl acetate extract		Trolox (positive control)	Ascorbic acid (positive control)
	ABTS free radical scavenging activity (%)	DPPH free radical scavenging activity (%)	ABTS free radical scavenging activity (%)	DPPH free radical scavenging activity (%)
0.1	ND	4.388 ± 1.240****	22.901 ± 0.512	29.346 ± 1.395
0.2	0.426 ± 0.376****	9.681 ± 1.436****	27.548 ± 0.94	33.132 ± 1.120
0.3	2.228 ± 1.274****	13.199 ± 0.307****	33.096 ± 1.18	39.095 ± 0.703
0.4	7.633 ± 1.067****	17.420 ± 0.323****	37.648 ± 0.86	42.144 ± 0.614
0.5	11.332 ± 0.857****	20.703 ± 0.402****	41.062 ± 0.71	46.867 ± 0.588
0.6	16.453 ± 0.641****	25.025 ± 0.893****	45.851 ± 1.29	51.557 ± 1.314
0.7	22.569 ± 2.497****	28.241 ± 0.265****	49.786 ± 0.56	56.281 ± 0.531
0.8	26.695 ± 0.499****	31.959 ± 1.058****	54.196 ± 0.65	59.497 ± 0.758
0.9	31.389 ± 0.499****	34.907 ± 0.614****	58.226 ± 0.71	62.244 ± 0.760
1	35.798 ± 0.082****	39.899 ± 1.560****	63.442 ± 0.85	65.326 ± 1.019

Values expressed are means ± SD (*n* = 3). Symbol (****) indicates *p* < 0.0001 significant difference between ethyl acetate extract. SCJ and controls according to two-way analysis of variance (ANOVA) using Tukey’s multiple comparisons test. ND: not detected.

**Fig. 3:**
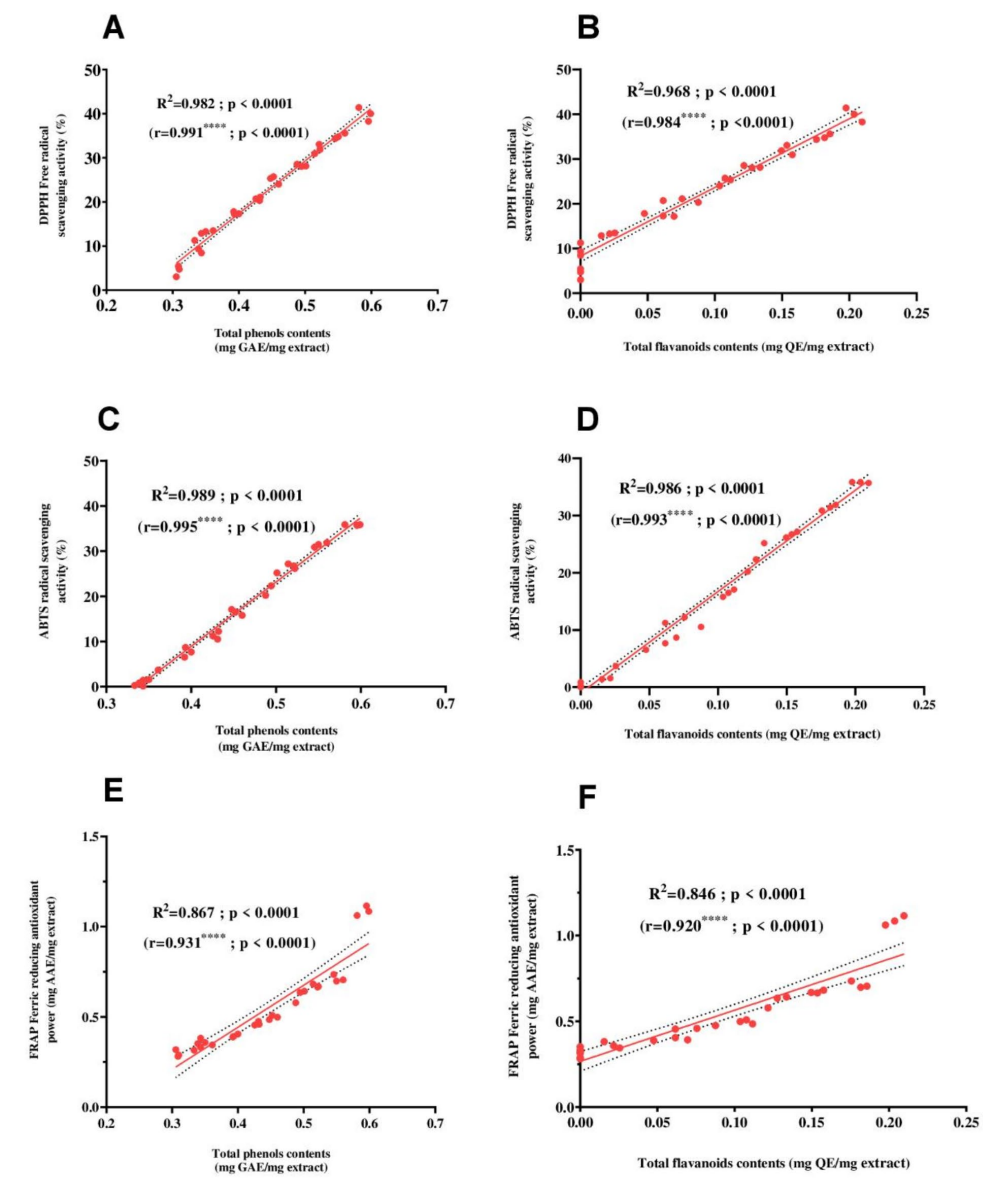
Pearson’s correlation coefficients between total phenolic and flavonoid contents and antioxidant activities ethyl acetate extract of *Streptomyces coeruleofuscus* SCJ. (**A**) Pearson correlation between DPPH and total phenolic contents, (**B**) Pearson correlation between DPPH flavonoid contents, (**C**) Pearson correlation between ABTS and total phenolic contents, (**D**) Pearson correlation between ABTS and flavonoid contents, (**E**) Pearson correlation between FRAP and total phenolic contents, and (**F**) Pearson correlation between FRAP and flavonoid contents. GAE: galic acid equivalent, QE: quercetin equivalent, AAE: ascorbic acid equivalent.

The statistical examination performed in this study supports this claim, indicating a significant correlation (*p* < 0.0001) between the antioxidant capacity of *Streptomyces coeruleofuscus* SCJ the ethyl acetate extract and its total phenolic and flavonoid contents. Figure 3 illustrated a strong positive correlation (*p* < 0.0001) between the polyphenol and flavonoid contents and the in vitro antioxidant activity of the extract. Significant positive correlations were found between the total phenolic contents and the DPPH assay (*r* = 0.991^****^, *p* < 0.0001), as well as between the total flavonoid contents and the DPPH assay (*r* = 0.984^****^, *p* < 0.0001). Additionally, remarkable positive correlations were observed between the total phenolic contents and the ABTS assay (*r* = 0.995, *p* < 0.0001), between the total flavonoid contents and the ABTS assay (*r* = 0.993^****^, *p* < 0.0001), between the total phenolic contents and the FRAP assay (*r* = 0.931^****^, *p* < 0.0001), and between the total flavonoid contents and the FRAP assay (*r* = 0.920^****^, *p* < 0.0001).

### Cytotoxicity of SCJ ethyl acetate extract on the triple-negative human breast carcinoma cell line (MDA-MB-468)

**Fig. 4:**
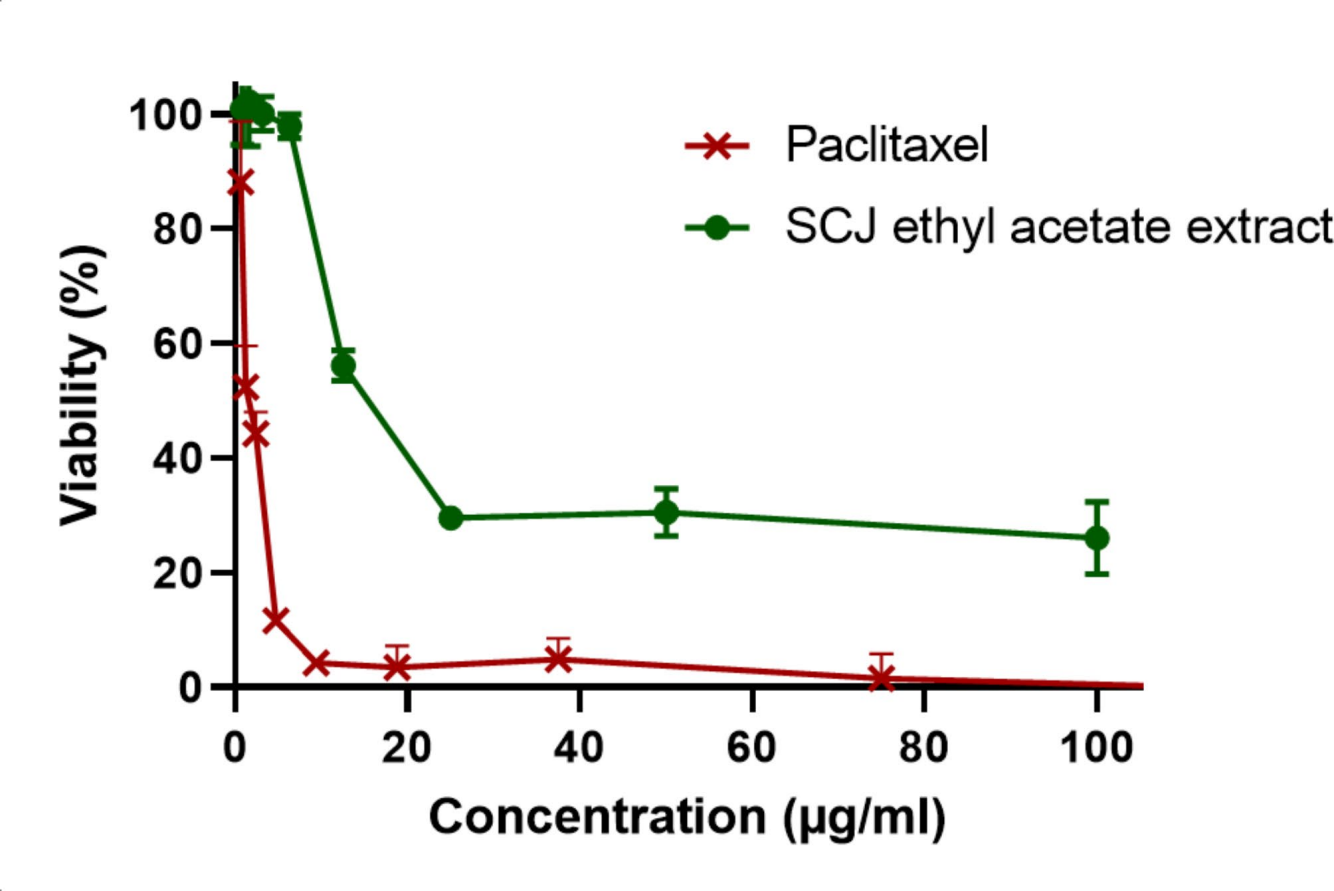
Cytotoxic Activity of Ethyl Acetate Extract from *Streptomyces coeruleofuscus* SCJ and the chemotherapeutic drug Paclitaxel, at varied Concentrations, on MDA-MB-468 Triple-Negative Breast Cancer Cells Following 24-Hour Treatment. The measurement of cell viability was done using crystal violet assay. All data are expressed as mean ± SD (*n* = 2).

The results of ethyl acetate extract of SCJ strain extract tested against the triple-negative breast cancer cell line (MDA-MB-468) are shown in Fig. 4. After a 24-hour treatment period, the ethyl acetate extract from the SCJ strain exhibited dose-dependent cytotoxic activity against the MDA-MB-468 triple-negative breast cancer cell line. It demonstrated an IC50 value of 11.3 ± 0.97 µg/mL, while the chemotherapeutic drug Paclitaxel displayed an IC50 value of 1.12 ± 0.43 µg/mL. Oxidative stress could drive cancer progression by modifying biological molecules and increasing mutation rates^47^. The growth inhibitory activity of the *Streptomyces coeruleofuscus* SCJ ethyl acetate extract was observed in cancer cells of the triple-negative human breast carcinoma cell line (MDA-MB-468).

### Exploring the anticancer potential of *Streptomyces coeruleofuscus* SCJ: insights into cytotoxic and antioxidant compounds

The secondary metabolites of *Actinobacteria*, particularly those of the *Streptomyces* genus, could serve as a significant source of potential new anticancer agents^48^. Retamycin, for example, aids in the treatment of human leukemia^49^, while geldanamycin is highly cytotoxic against human nasopharyngeal squamous cell carcinoma and breast cancer^50^. Yip et al.^48^ reported that *Streptomyces* sp. strain H7372.31-2 induced a cytostatic (antiproliferative) effect on MCF-7 and MDA-MB-231 breast cancer cells without inducing cytotoxicity (cell death). Hence, our findings are particularly intriguing, revealing that *Streptomyces coeruleofuscus* SCJ elicits significant cytotoxicity, demonstrating a substantial inhibition rate of about 25% at 100µg/mL against the MDA-MB-468 cancer cell line. Moreover, regarding the ethyl acetate extract from the SCJ strain used in this study, our previous work^33^ showed that GC-MS analysis of the extract identified compounds with high biological activities. Among the compounds identified were Piperazine derivatives such as “Piperazine, 1-methyl-4-[2-(p-tolylsulfonyl) ethyl]-” and pyrrolopyrazine derivatives such as “Pyrrolo[1,2-a] pyrazine 1,4-dione, hexahydro-3-(2-methylpropyl)-“. Piperazines are heterocyclic compounds found in nature and generally produced by microorganisms^51^. Moreover, piperazines are characterized by typical volatile and odorous metabolites produced by *Streptomyces*^52,53^. many piperazines has been associated with various pharmacological activities such as antitumor, anticonvulsant, antidepressant, analgesic, antimicrobial, antitubercular, antidiabetic, antihistaminic, anti-inflammatory and others^54^. Additionally, pyrrolizidine can be found and produced by *Streptomyces*^55^. Furthermore, pyrrolizidine is recognized for its diverse bioactivities, such as antitumor, anti-angiogenesis, and antioxidant properties^55,56^. For instance, the identification of the compound “Pyrrolo[1,2-a] pyrazine 1,4-dione, hexahydro-3-(2-methylpropyl)-” in the ethyl acetate extract of the SCJ strain implies that its antioxidant activity might be attributed to this particular compound. Moreover, this compound was effectively purified from Bacillus sp. associated with a marine sponge, demonstrating a noteworthy antioxidant effect capable of mitigating cellular oxidative damage induced by oxygen free radicals^57^. The compounds of pyrrolizidine have also been linked to promising anticancer activity^40^. The results of this study suggest that these heterocyclic compounds could have been responsible for the antioxidant and cytotoxic activities of ethyl acetate extract of the SCJ strain against TNBC. Another compound, “3,5-Dimethylpyrazole’’, containing a pyrazole nucleus, was found in the ethyl acetate extract of the SCJ strain. The presence of this pyrazole nucleus in different structures leads to diverse applications in different fields such as technology, medicine and agriculture. These include protein glycation inhibitors, antibacterial agents, antifungals, anticancer agents, antidepressants, anti-inflammatories, antituberculosis agents, antioxidants and antivirals^58,59^.

### HPLC-UV/vis analysis of SCJ ethyl acetate extract

**Fig. 5:**
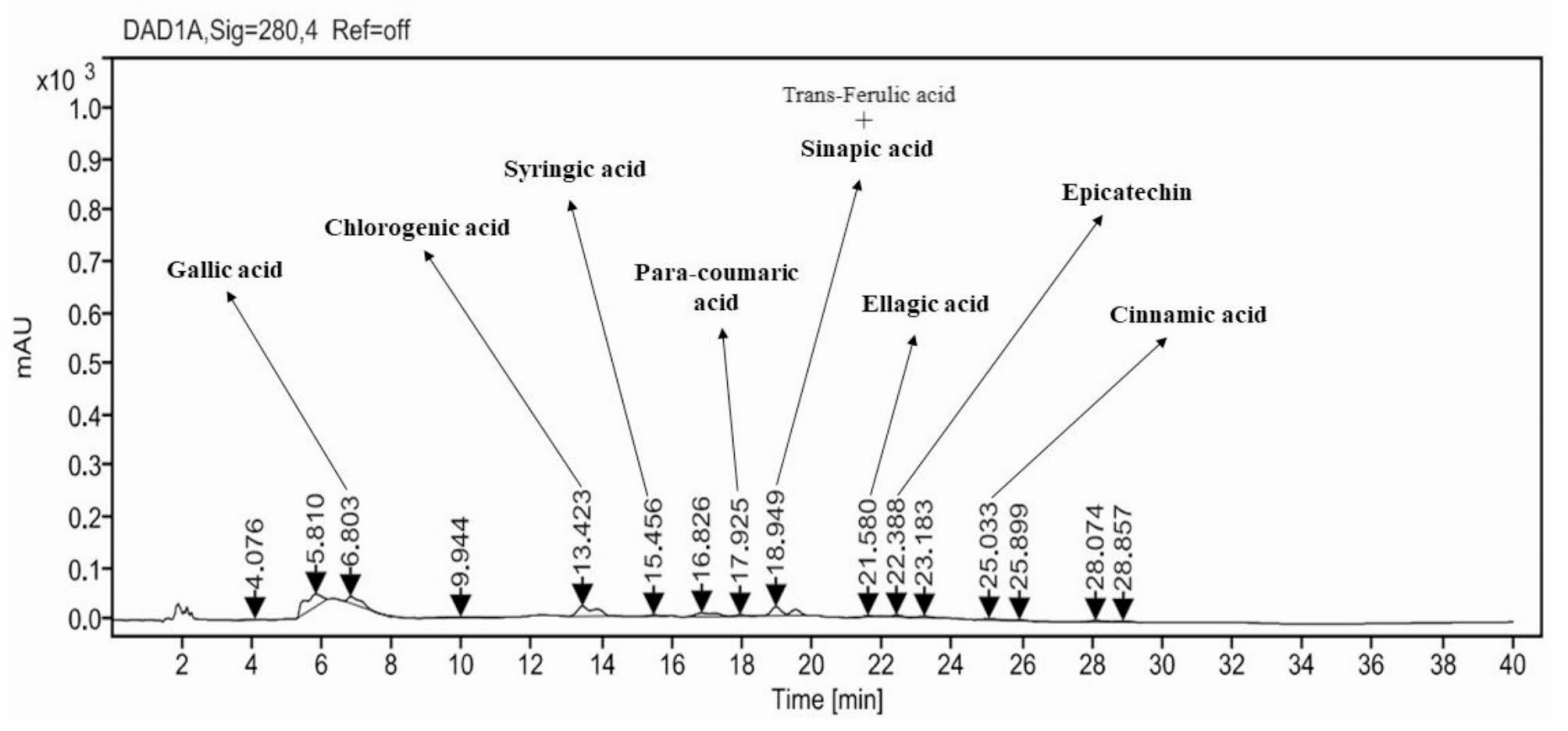
HPLC chromatogram of the crude ethyl acetate extract of *Streptomyces coeruleofuscus* SCJ.

The analysis of the ethyl acetate extract from *Streptomyces coeruleofuscus* SCJ using HPLC-UV/Vis revealed the presence of nine distinct phenolic compounds, including gallic acid, sinapic acid, *p-*Coumaric acid, cinnamic acid, trans-ferulic acid, syringic acid, chlorogenic acid, ellagic acid and epicatechin (Fig. 5; Table 3). These compounds, in addition to their antioxidant capacity to prevent damage caused by oxidative stress^60^, are known to modify epigenetic mechanisms such as chromatin remodeling^61^. These modifications influence gene expression and play an important role in cancer prevention and treatment by acting on the processes involved in tumor development^62^. For example, gallic acid (GA) possesses anti-cancer properties, acting in two primary ways. Firstly, it regulates oxidative stress, reducing cellular damage that can lead to cancer. Secondly, it alters the oxidoreductive state of cancer cells, disrupting their metabolism and function, which can slow their growth or lead to their destruction^63,64^. It has been reported that gallic acid (GA) inhibits the proliferation of various types of cancer cells, including those associated with leukemia, breast cancer, prostate cancer, cervical cancer, lung cancer, stomach cancer, colon cancer, brain cancer, melanoma, and esophageal cancer^65–67^. Similarly, ellagic acid demonstrates anti-carcinogenic effects by inducing apoptosis in cancer cells and inhibiting their proliferation^68,69^. Additionally, sinapic acid has demonstrated important anti-cancer potential in several studies. Proposed as a potent antioxidant, it has shown chemo-preventive effects against colon cancer in rat models^70^. Research also revealed its ability to inhibit the growth of human prostate cancer cell lines (PC-3 and LNCaP) by modulating apoptosis-related proteins such as BAX, CASP3, CASP8, and CYCS, while upregulating FAS, TIMP-1, CDH1, and downregulating MMP-9^71^. Moreover, sinapic acid exhibited cytotoxic effects against various cancer cell lines, including human laryngeal carcinoma (HEp-2)^72^, Chinese hamster lung fibroblasts (V79), human cervical carcinoma (HeLa)^73^, and breast cancer cell lines MCF7, MDA-MB-231^74^, and T47D^75^. These findings suggest that sinapic acid is a promising bioactive compound in cancer therapy. In a study conducted by Kanski et al. 2002^76^. As for ferulic acid (FA), its antioxidant potential has been demonstrated through its ability to reduce neuronal damage caused by reactive oxygen species (ROS). Additionally, FA inhibited the proliferation of certain cancer cells, including T47D breast cancer cells and ECV304 endothelial cells^75,77^. In Caco-2 cells, FA treatment led to an extension of the S phase and altered the distribution of cell cycle phases^78^. In parallel, *p*-coumaric acid reduced the viability of HCT-15 and HT-29 cells at concentrations of 1400 and 1600 µmol/L, respectively^79^. The antiproliferative effect of *p-*coumaric acid was further demonstrated by a significant decrease in the number of colonies formed after 72 h of treatment, with 32 colonies for HCT-15 cells and 51 for HT-29 cells, compared to 105 and 154 colonies in untreated cells^79^. These findings confirm the effectiveness of *p*-coumaric acid as an anticancer agent^79^. Furthermore, Pal et al. (2020)^80^ demonstrated that cinnamic acid has an antiproliferative effect on triple-negative breast cancer cells (MDA-MB-231) and tumorigenic human embryonic kidney cells (HEK293), while sparing normal cells such as NIH3T3 fibroblasts. This suggests that cinnamic acid may specifically target cancer cells without affecting healthy tissues. Syringic acid (SA) has demonstrated promising properties in combating certain cancers. Although it does not affect mammalian polymerase activity, studies have shown its antiproliferative potential. Phytochemical extraction of SA from proso millet (Panicum miliaceum L.) revealed that it partially inhibits the proliferation of cancer cells, specifically human breast cancer (MDA) and liver cancer (HepG2) cells^81^. In T47D breast cancer cells, SA reduced cell proliferation by 20% with an IC50 of less than 10^− 12^ M, indicating significant inhibitory potential^75^. Regarding chlorogenic acid, chlorogenic acid (CGA), a polyphenolic compound, it possesses various biological activities, including anticancer, antibacterial, and antioxidant effects^82–84^. In the study by Zeng et al. (2021), CGA was identified as a potent inhibitor of the NF-κB/EMT signaling pathway, exhibiting significant antitumor activity in breast cancer^85^. Finally, it has been shown that epicatechin, a flavonoid belonging to a large family of biomolecules, reduces the metastatic process in a murine model of triple-negative breast cancer (4T1 cells) by decreasing molecular markers associated with metastasis^86^. These findings suggest that epicatechin could represent a promising therapeutic approach, potentially serving as an adjuvant to enhance the efficacy of anticancer treatments. Numerous studies have shown that the synergy between polyphenols can improve the efficacy of cancer treatment compared to the administration of a single polyphenol^87,88^. Recent data indicate that this phenolic synergy can significantly inhibit cell growth and induce apoptosis in MDA-MB-231 cells, a model of triple-negative breast cancer (TNBC)^87^. Indeed, the synergistic effect of the combined polyphenols exceeds the efficacy of each polyphenol administered individually, suggesting that this therapeutic approach is more promising for treating this aggressive type of breast cancer.

**Table 3:** The qualitative analysis of phenolic compounds in the ethyl acetate extract of *Streptomyces coeruleofuscus* SCJ using HPLC-UV/visible.

Standard number	Standard name	Retention time (min)	Ethyl acetate extract
1	Gallic acid	6.4	6.8
2	Vanillin	16.2	ND
3	Ferulic acid	18.3	ND
4	Vanillic acid	14.1	ND
5	Sinapic acid	18.2	18.9
6	*p*-coumaric acid	17.7	17.9
7	Caffeic acid	14.7	ND
8	Cinnamic acid	24.6	25
9	Quercetin	26.6	ND
10	Trans-ferulic acid	18.5	18.9
11	Syringic acid	15.4	15.4
12	Chlorogenic acid	13.3	13.4
13	Ellagic acid	21.9	21.5
14	Epicatechin	22.2	22.3
15	Resorcinol	8.5	ND

ND: not detected.

### In silico prediction of ADMET properties

A major impediment to drug development in clinical research is attributed to unfavorable pharmacokinetic characteristics, the limited ADMET characteristics of a drug compromise its effectiveness and can significantly increase costs^89^. In the present study, we used in silico approaches as a cost and time-effective method to rapidly screen pharmacokinetic and toxicity properties of compounds identified in ethyl acetate crude extract of *Streptomyces coeruleofuscus* SCJ. The results of this study are detailed in Tables 4 and 5.

**Table 4:** ADMET properties of the volatile’s compounds identified in ethyl acetate extract of *Streptomyces coeruleofuscus* SCJ.

	Compounds		1	2	3	4	5	6	7	8	9
**Drug-Likeness**	Lipinski 5 rules		Yes	Yes	Yes	Yes	Yes	Yes	Yes	Yes	Yes
	Bioavailability Score		0.55	0.85	0.55	0.55	0.55	0.55	0.55	0.55	0.55
**Absorption**	Water solubility (Log mol/L)		-1.24	-3.51	-1.00	-1.39	-5.31	-1.07	-3.56	-3.32	-2.06
	Caco2 permeability (Log Papp; log cm/s)		1.39	1.35	1.58	1.53	1.74	1.49	1.63	1.60	1.19
	Intestinal absorption (%)		95.18	94.60	97.44	79.46	93.65	97.22	92.47	94.8	84.24
	Skin Permeability Log Kp		-2.17	-2.36	-2.87	-2.82	-2.42	-2.54	-1.93	-1.81	-3.97
	P-glycoprotein substrate		Yes	No	No	Yes	No	Yes	No	No	No
	P-glycoprotein I inhibitor		No	No	No	No	Yes	No	No	No	No
	P-glycoprotein II inhibitor		No	No	No	No	No	No	No	No	No
**Distribution**	VDss (human)		0.01	-0.41	-0.01	1.18	0.26	0.11	-0.06	0.25	0.10
	Fraction unbound (human)		0.69	0.03	0.75	0.62	0.15	0.68	0.40	0.4	0.6
	BBB permeability (log BB)		0.16	-0.10	-0.41	0.05	-0.14	0.14	0.52	0.61	-0.12
	CNS permeability (log PS)		-2.49	-2.26	-3.04	-2.70	-2.26	-2.73	-2.50	-2.32	-2.99
**Metabolism**	CYP2D6 substrate		No	No	No	No	No	No	No	No	No
	CYP3A4 substrate		No	Yes	No	No	Yes	No	No	No	No
	CYP2D6 inhibitior		No	No	No	No	No	No	No	No	No
	CYP3A4 inhibitior		No	No	No	No	No	No	No	No	No
**Excretion**	Total Clearance (log mL/min/kg)		0.33	-0.24	0.59	0.62	1.63	0.50	0.59	1.42	1.21
	Renal OCT2 substrate		No	No	No	No	No	No	No	No	No
**Toxicity**	AMES toxicity		No	No	No	No	No	No	No	No	No
	Max. tolerated dose (human) (log mg/kg/day)		1.01	1.1	0.60	-0.04	0.06	0.95	0.43	0.41	0.62
	hERG I inhibitor		No	No	No	No	No	No	No	No	No
	hERG II inhibitor		No	No	No	No	No	No	No	No	No
	Oral Rat Acute Toxicity (LD50) (mol/kg)		2.66	2.31	2.21	2.62	1.84	2.02	2.68	1.83	2.03
	Oral Rat Chronic Toxicity (log mg/kg_bw/day)		1.62	1.30	1.67	1.80	2.42	1.82	0.78	2.37	2.26
	Hepatotoxicity		No	No	No	Yes	No	No	No	No	No
	Skin Sensitisation		No	No	Yes	No	Yes	Yes	Yes	Yes	No
	T.Pyriformis toxicity (log ug/L)		0.17	1.34	-0.48	0.27	2.09	-0.55	2.12	0.80	0.30
	Minnow toxicity (log mM)		1.93	0.37	2.71	1.47	-0.34	2.24	0.85	0.61	2.46

(1): Disulfide, dimethyl ; (2): Phosphonic acid, diphenyl ester ; (3): 3,5-Dimethylpyrazole ; (4): Piperazine, 1-methyl-4-[2-(p-tolylsulfonyl) ethyl] - ; (5): Tetrahydropyran Z-10-dodecenoate ; (6): 2 H-Pyran, tetrahydro-2-methyl- ; (7): Trifluoroacetyl-lavandulol ; (8): 2(3 H) -Furanone, 5-heptyldihydro- ; (9): Pyrrolo[1,2-a] pyrazine 1,4-dione, hexahydro-3-(2 methylpropyl) – .

**Table 5:** ADMET properties of compounds identified by HPLC in the ethyl acetate extract of *Streptomyces coeruleofuscus* SCJ.

	Compounds		10	11	12	13	14	15	16	17	18
**Drug-Likeness**	Lipinski 5 rules		Yes	Yes	Yes	Yes	Yes	Yes	Yes	Yes	Yes
	Bioavailability Score		0.56	0.56	0.85	0.85	0.85	0.56	0.11	0.55	0.55
**Absorption**	Water solubility (Log mol/L)		-0.72	-2.27	-1.46	-1.49	-1.84	-1.79	-3.50	-3.94	-3.49
	Caco2 permeability (Log Papp; log cm/s)		-0.46	0.35	1.19	1.78	0.26	0.28	-0.06	-0.11	-0.22
	Intestinal absorption (%)		50.31	92.82	92.91	92.83	92.87	66.16	59.60	82.76	78.47
	Skin Permeability Log Kp		-3.08	-3.06	-2.78	-2.42	-2.94	-3.07	-3.25	-3.56	-3.67
	P-glycoprotein substrate		Yes	Yes	Yes	Yes	Yes	Yes	Yes	Yes	Yes
	P-glycoprotein I inhibitor		No	No	No	No	No	No	No	No	No
	P-glycoprotein II inhibitor		No	No	No	No	No	No	No	No	No
**Distribution**	VDss (human)		-1.07	-0.15	-0.10	0.011	-0.12	-0.24	-0.43	-0.20	-0.05
	Fraction unbound (human)		0.565	0.495	0.512	0.509	0.504	0.519	0.496	0.429	0.435
	BBB permeability (log BB)		-0.93	-0.24	-0.22	0.25	-0.22	-0.23	-0.86	-0.64	-0.32
	CNS permeability (log PS)		-2.81	-2.63	-2.39	-1.62	-2.51	-2.63	-3.23	-2.47	-2.41
**Metabolism**	CYP2D6 substrate		No	No	No	No	No	No	No	No	No
	CYP3A4 substrate		No	No	No	No	No	No	Yes	Yes	No
	CYP2D6 inhibitior		No	No	No	No	No	No	No	No	No
	CYP3A4 inhibitior		No	No	No	No	No	No	No	No	No
**Excretion**	Total Clearance (log mL/min/kg)		0.55	0.357	0.377	0.389	0.396	0.325	0.432	0.714	0.779
	Renal OCT2 substrate		No	No	No	No	No	No	No	No	No
**Toxicity**	AMES toxicity		No	Yes	Yes	No	Yes	Yes	Yes	Yes	Yes
	Max. tolerated dose (human) (log mg/kg/day)		1.404	1.38	1.624	1.689	1.502	1.493	0.635	0.282	0.547
	hERG I inhibitor		No	No	No	No	No	No	No	No	No
	hERG II inhibitor		No	No	No	No	No	No	No	No	No
	Oral Rat Acute Toxicity (LD50) (mol/kg)		1.872	2.235	1.965	1.892	2.105	2.191	2.004	2.232	2.39
	Oral Rat Chronic Toxicity (log mg/kg_bw/day)		1.499	1.555	2.882	2.764	2.753	1.566	1.554	1.376	1.501
	Hepatotoxicity		No	No	No	No	No	No	No	No	No
	Skin Sensitisation		No	No	No	Yes	No	No	No	No	No
	T.Pyriformis toxicity (log ug/L)		-0.07	1.011	0.005	0.011	0.548	0.54	0.685	0.454	1.147
	Minnow toxicity (log mM)		2.918	1.601	1.696	1.59	1.649	1.766	2.241	2.131	1.796

(10): Gallic acid ; (11): Sinapinic acid ; (12): *p*-coumaric acid ; (13): Cinnamic acid ; (14): Trans-Ferulic acid ; (15): Syringic acid ; (16): Chlorogenic acid ; (17): Ellagic acid ; (18): Epicatechin.

**Fig. 6:**
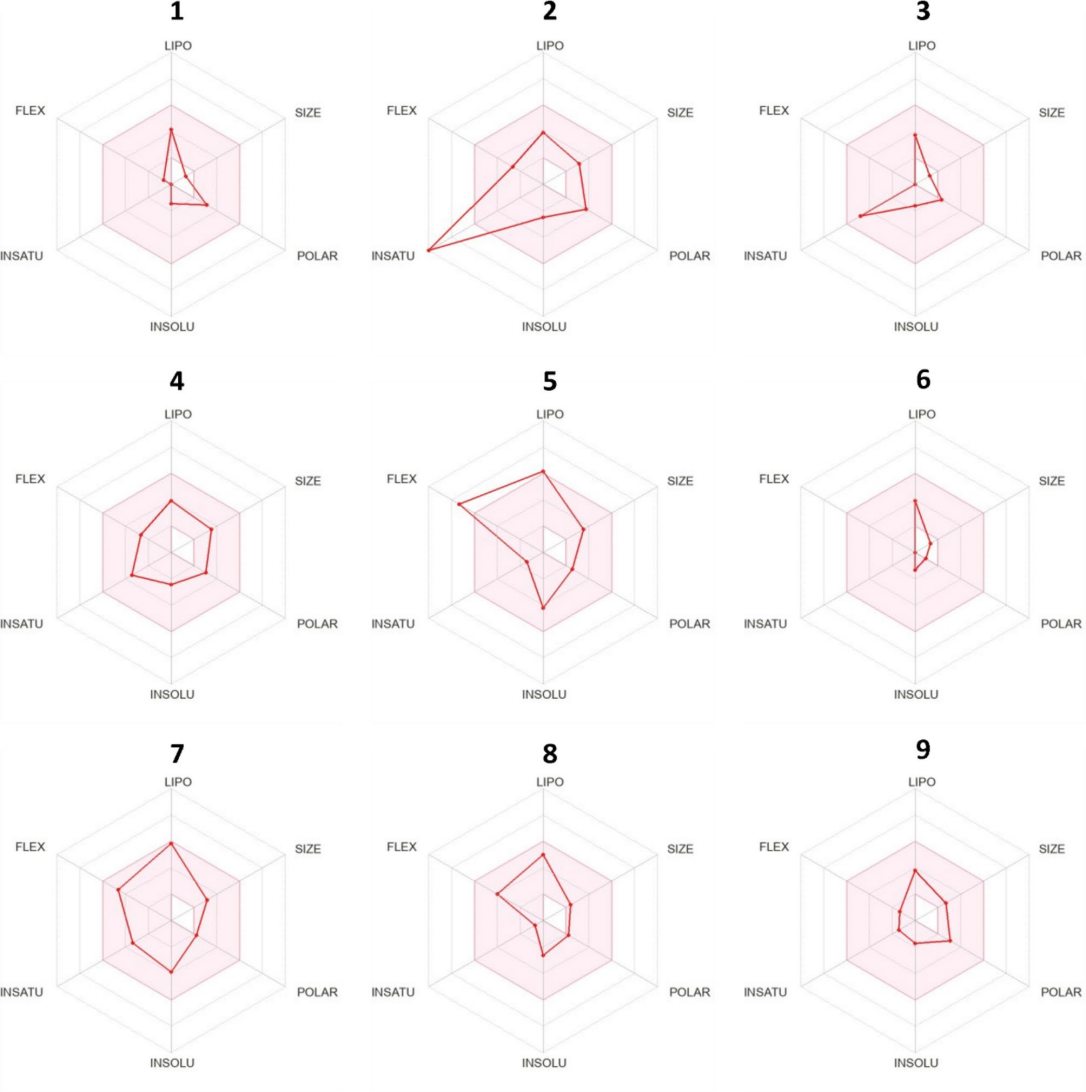
Bioavailability radars of volatile compounds identified from the ethyl acetate crude extract of *Streptomyces coeruleofuscus* SCJ considering six physicochemical properties (lipophilicity, size, polarity, solubility, flexibility and saturation).

**Fig. 7:**
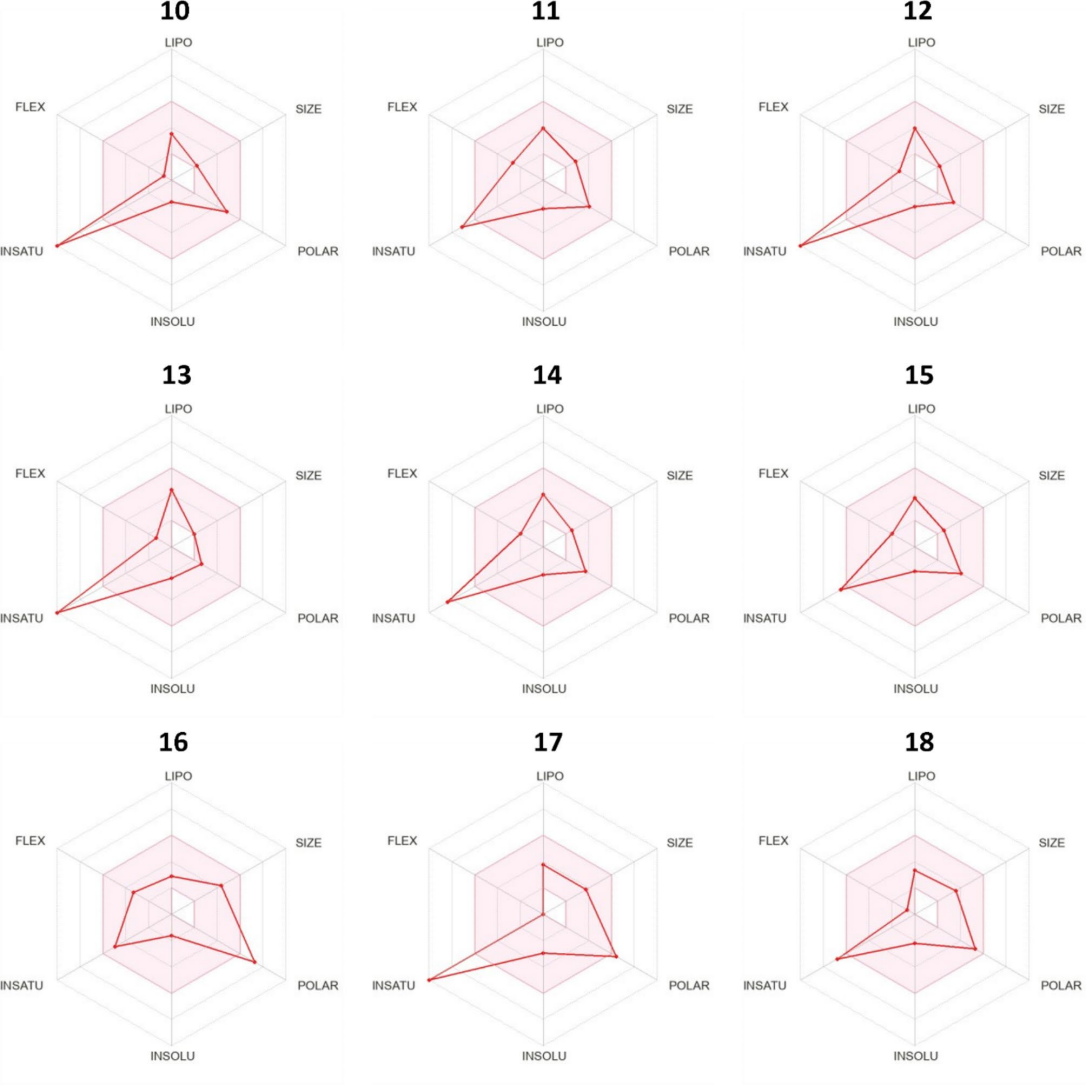
Bioavailability radars of compounds identified by HPLC from the ethyl acetate crude extract of *Streptomyces coeruleofuscus* SCJ considering six physicochemical properties (lipophilicity, size, polarity, solubility, flexibility and saturation).

The findings indicate that all identified compounds exhibit drug-like properties and comply with Lipinski’s rule of five (hydrogen bond donors < 5, hydrogen bonds acceptors < 10, N or O ≤ 10, MW < 500 Da, and MLOGP ≤ 5). Moreover, most molecules displayed promising bioavailability, registering at 0.55, except for compounds 2, 12, 13 and 14 which show a bioavailability of 0.85, while compound 16 has low bioavailability, estimated at 0.11 (Tables 4 and 5). Additionally, they demonstrated good water solubility with a logS ranging from − 4 to 0. Figures 6 and 7 illustrates the compounds bioavailability radars,. where the pink zone represents the ideal oral bioavailability space. In this investigation, all compounds conform to the designated space for oral bioavailability, except for molecules 2, 10, 12, 13, 14 and 17, which exhibit high saturation levels, and molecules 5 and 16, which exhibit high flexibility and polarity, respectively, excluding them from this acceptable zone.

Regarding the absorption properties, volatile compounds showed high Caco-2 permeability (> 0.90), but only compounds 12 and 13 identified by HPLC display good permeability. A molecule is considered poorly absorbed when its absorption rate is below 30%. In this study, the intestinal absorption rate of the compounds ranges from 50.31 to 97.44%. Additionally, the permeability coefficient (Kp), which measures a compound’s ability to cross mammalian skin, is critical for drugs intended for topical use. A more negative log Kp indicates lower skin permeability. Our compounds showed log Kp values ranging from − 3.93 to -1.81. Furthermore, volatile compounds 1, 4, and 6, as well as all the compounds identified by HPLC, could serve as substrates for P-glycoprotein, while compound 5 is the only predicted inhibitor of P-glycoprotein I.

The steady-state volume of distribution (VDss, expressed in log L/kg) reflects the degree of drug distribution in the body. A VDss greater than 0.45 is considered high, while a value below − 0.15 is considered low. Our compounds showed varying VDss values: compound 4 exhibited the highest VDss (1.182), while compound 16 showed the lowest (-0.43). Most other compounds showed moderate values, ranging from − 0.05 to 0.263. These results suggest that most constituents of the *Streptomyces coeruleofuscus* SCJ extract have substantial distribution capacity, particularly compound 16, aiding in achieving therapeutic concentrations in blood plasma.

The blood-brain barrier (BBB), which protects the brain from foreign substances, is a crucial factor in evaluating distribution properties. Our results, presented in Tables 4 and 5, show that the studied compounds have good permeability across the BBB, making them promising candidates for treating brain cancers and metastases.

Concerning metabolism, it is important to analyze the interactions between compounds and cytochrome P450, particularly their roles as inhibitors or substrates of the CYP2D6 and CYP3A4 isoenzymes, which are essential for drug metabolism. None of the identified compounds inhibit these enzymes, but compounds 2, 5, 16, and 17 are potential substrates for CYP3A4. Additionally, none of the compounds were found to be substrates of the renal transporter OCT2. Compound 5 shows the highest total clearance with a value of 1.63 log(mL/min/kg).

The toxicological evaluation of the compounds was performed through various tests, including the AMES test, maximum tolerated dose, hERG inhibition, acute and chronic oral toxicity in rats, hepatotoxicity, and skin sensitization. As shown in Table 4, none of the volatile molecules examined exhibit mutagenic or carcinogenic properties according to the AMES test, although this test was unfavorable for the majority of compounds identified by HPLC, except for molecules 10 and 13. Furthermore, none of the compounds showed hERG inhibition or hepatotoxicity, except for compound 4, which was predicted to be potentially hepatotoxic. On the other hand, the volatile compounds 1, 2, 4, and 9, as well as all compounds identified by HPLC except compound 13, are unlikely to induce skin sensitization, unlike the other compounds, which are deemed unsuitable for topical use.

### Exploration of human target receptors

The results obtained from the Swiss Target Prediction tool have unveiled that the 9 volatile compounds hold the potential to interact with hundreds of different human receptors. Our findings emphasize specific targets, focusing on their roles in the regulation of cell survival and death. Notably, the estrogen receptor beta (ER beta) emerges as a common target for the majority of the examined compounds. Despite the absence of estrogen receptors being a characteristic of triple-negative breast cancer (TNBC), recent research indicates that ER beta is present in over half of TNBC cases and in approximately 20 to 30% of all breast cancers. Some studies even propose ER beta as a potential therapeutic target for TNBC treatment^90,91^. Furthermore, the presence of various targets such as mitogen-activated protein kinase 2, P38 alpha, c-Jun N-terminal kinase 3, Cytochrome P450 19A1, and X-linked inhibitor of apoptosis protein 3 (XIAP) — all implicated in cell survival and death — suggests that these compounds may exert a synergistic effect, contributing to the observed cytotoxic activity of the *Streptomyces coeruleofuscus* SCJ ethyl acetate crude extract on MDA-MB-468 breast cancer cells.

### Molecular docking interactions

**Fig. 8:**
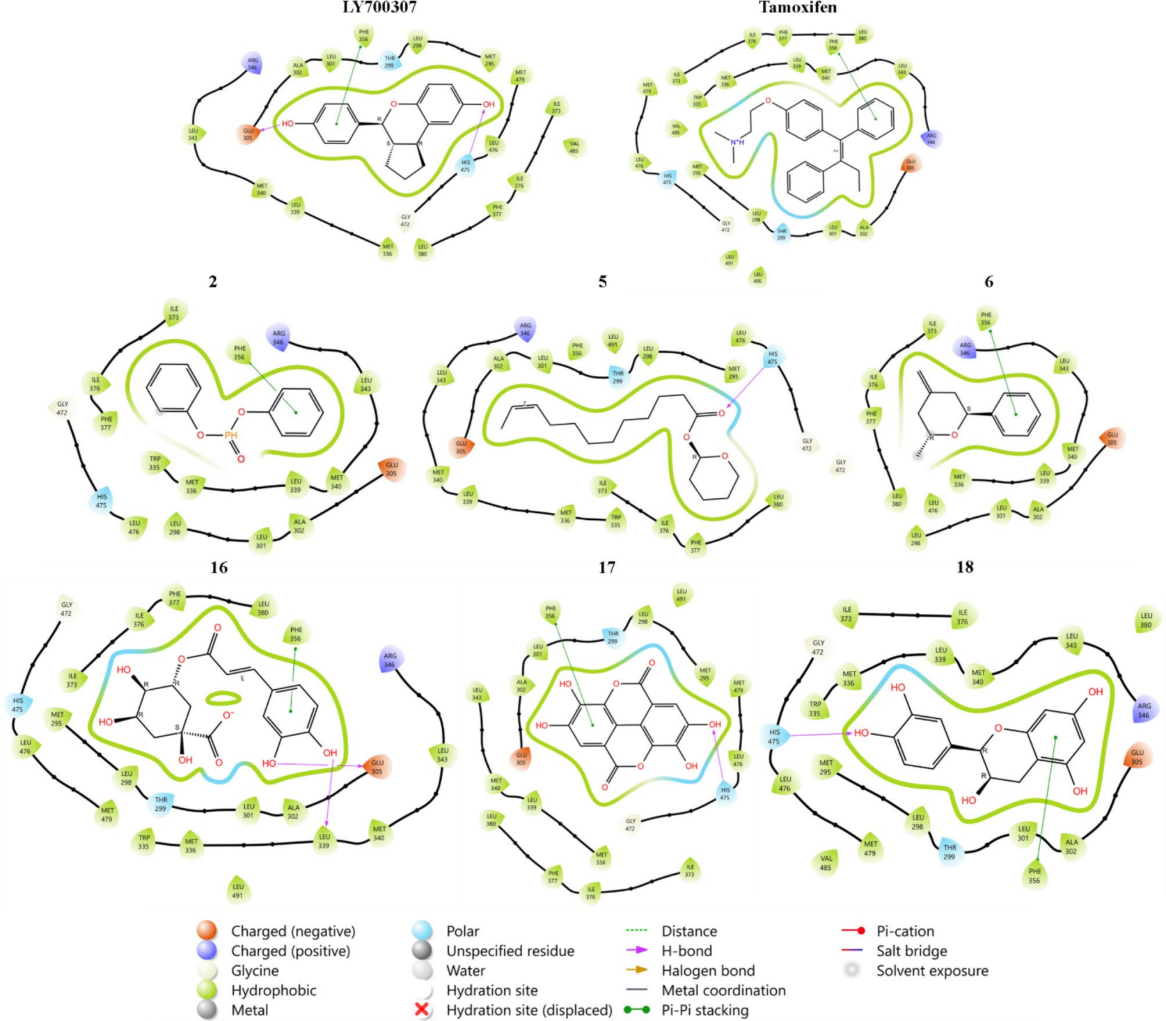
2D Molecular docking interactions of the identified compounds with the most active energy (> 7.0 Kcal/mol) and two reference ligands (LY700307 and Tamoxifen), with Human Estrogen Receptor Beta Ligand-binding Domain in Complex with (R)-2-(2-chloro-4-hydroxyphenyl)-3-(4-hydroxyphenyl) propanenitrile (PDB: 7XWQ), (resolution: 1.89 Å), root mean square deviation (RMSD) < 1.

During this study, molecular docking results revealed that among all analyzed molecules, six exhibited significant affinity towards the estrogen receptor beta (ERβ), with a binding free energy exceeding − 7.0 kcal/mol (Table 6). It was found that this energy value is comparable to that of Tamoxifen, with a binding energy of -5.1 kcal/mol, which has been reported as a ligand that binds to ERβ^92^. Additionally, it was found that the results reported by Mukherjee and Majumder were consistent with those of our results, indicating similar findings in terms of molecular docking energy^93^. Consequently, we hypothesize that the identified molecules may have an improved effect compared to Tamoxifen. Furthermore, exploring the individual implications of active site residues in the ligand-receptor interaction (Fig. 8) highlighted the importance of PHE356 in the binding of the synthetic ligand LYD700307 to ERβ with an energy of -10.2 kcal/mol, explaining their high selectivity in various experimental models and promising effects on proliferation, inhibition, and suppression of invasiveness^90^. Indeed, we suggest that the aromatic cycle of spotted compounds, bound to the PHE356 residue through Pi-Pi stacking interactions (Fig. 8), may have a primary effect on the notable binding energy of these compounds and could be considered as potential promoters of ERβ inhibitors. However, to achieve reliable and comprehensive results, further studies should delve into investigations using cell lines expressing estrogen receptor beta.

**Table 6:** Docking results of the various compounds identified in the ethyl acetate crude extract of *Streptomyces coeruleofuscus* SCJ with human estrogen receptor *Beta.*

Compounds	Affinity energy (Kcal/mol)	Compounds	Affinity energy (Kcal/mol)
LYD700307	-10.2	10	-5.8
Tamoxifen	-5.1	11	-6.4
1	-2.1	12	-6.2
2	-7.5	13	-6.0
3	-4.1	14	-6.3
4	-7.0	15	-5.8
5	-7.5	16	-7.7
6	-7.7	17	-8.3
7	-6.7	18	-8.3
8	-5.9		
9	-6.8		

(1): Disulfide, dimethyl ; (2): Phosphonic acid, diphenyl ester ; (3): 3,5-Dimethylpyrazole ; (4): Piperazine, 1-methyl-4-[2-(p-tolylsulfonyl) ethyl] - ; (5): Tetrahydropyran Z-10-dodecenoate ; (6): 2 H-Pyran, tetrahydro-2-methyl- ; (7): Trifluoroacetyl-lavandulol ; (8): 2(3 H) -Furanone, 5-heptyldihydro- ; (9): Pyrrolo[1,2-a] pyrazine 1,4-dione, hexahydro-3-(2 methylpropyl) –. (10): Gallic acid ; (11): Sinapinic acid ; (12): *p*-coumaric acid ; (13): Cinnamic Acid ; (14): Trans-Ferulic Acid ; (15): Syringic acid ; (16): Chlorogenic acid ; (17): Ellagic acid ; (18): Epicatechin.

## Conclusions

This study demonstrated that *Streptomyces coeruleofuscus* SCJ, isolated from Moroccan garden soil, is a promising source of secondary metabolites with significant antioxidant and anticancer activities. The ethyl acetate extract showed potent antioxidant properties by reducing DPPH and ABTS radicals and chelating iron in the FRAP assay, likely due to the presence of phenolic and flavonoid compounds. Additionally, the extract exhibited cytotoxicity against triple-negative human breast carcinoma cells (MDA-MB-468). the identified compounds demonstrated favorable pharmacokinetic properties and potential affinity with the estrogen receptor beta (ERβ), indicating their therapeutic potential for TNBC. Future research should focus on experimental validation of these compounds’ activity, particularly in ERβ-expressing cell lines, along with further pharmacodynamic and pharmacokinetic studies to elucidate their mechanism of action and in vivo efficacy, paving the way for new therapeutic strategies against triple-negative breast cancer. Exploring synergies between the compounds and conducting molecular dynamics simulations could further enhance the understanding and development of targeted therapies. These findings underscore the potential of *Streptomyces coeruleofuscus* SCJ in contributing to drug discovery efforts.

## Data Availability

Genomic sequence of Streptomyces coeruleofuscus SCJ has been deposited at the National Centre for Biotechnology Information (NCBI) GenBank (https://www.ncbi.nlm.nih.gov/nucleotide/) under the following accession number OP101646.
